# Comparison of Standing Posture Bioelectrical Impedance Analysis with DXA for Body Composition in a Large, Healthy Chinese Population

**DOI:** 10.1371/journal.pone.0160105

**Published:** 2016-07-28

**Authors:** Kuen-Tsann Chen, Yu-Yawn Chen, Chia-Wei Wang, Chih-Lin Chuang, Li-Ming Chiang, Chung-Liang Lai, Hsueh-Kuan Lu, Gregory B. Dwyer, Shu-Ping Chao, Ming-Kuei Shih, Kuen-Chang Hsieh

**Affiliations:** 1 Department of Applied Math, National Chung Hsing University, Taichung, Taiwan; 2 Department of Physical Education, National Taiwan University of Sport, Taichung, Taiwan; 3 Department of Cosmetic Application & Management, St. Mary's Junior College of Medicine, Nursing and Management, Ilan, Taiwan; 4 Department of Radiology, Jen-Ai Hospital, Taichung, Taiwan; 5 Department of Hospitality, Recreation, and Tourism Management, East Stroudsburg University, Pennsylvania, United States of America; 6 Department of Physical Medicine and Rehab, Taichung Hospital, Ministry of Health and Welfare, Taichung, Taiwan; 7 Sport Science Research Center, National Taiwan University of Sport, Taichung, Taiwan; 8 Department of Exercise Science, East Stroudsburg University, Pennsylvania, United States of America; 9 Department of Exercise Health Science, National Taiwan University of Sport, Taichung, Taiwan; 10 Department of Food and Beverage management, National Kaohsiung University of Hospitality and Tourism, Kaohsiung, Taiwan; 11 Fundamental Education Center, National Chin-Yi University of Technology, Taichung, Taiwan; 12 Research Center, Charder Electronic Co., Ltd, Taicung, Taiwan; University of Sydney, AUSTRALIA

## Abstract

Bioelectrical impedance analysis (BIA) is a common method for assessing body composition in research and clinical trials. BIA is convenient but when compared with other reference methods, the results have been inconclusive. The level of obesity degree in subjects is considered to be an important factor affecting the accuracy of the measurements. A total of 711 participants were recruited in Taiwan and were sub-grouped by gender and levels of adiposity. Regression analysis and Bland-Altman analysis were used to evaluate the agreement of the measured body fat percentage (BF%) between BIA and DXA. The BF% measured by the DXA and BIA methods (Tanita BC-418) were expressed as BF%_DXA_ and BF%_BIA8_, respectively. A one-way ANOVA was used to test the differences in BF% measurements by gender and levels of adiposity. The estimated BF%_BIA8_ and BF%_DXA_ in the all subjects, male and female groups were all highly correlated (*r* = 0.934, 0.901, 0.916, all *P*< 0.001). The average estimated BF%_BIA8_ (22.54 ± 9.48%) was significantly lower than the average BF%_DXA_ (26.26 ± 11.18%). The BF%_BIA8_ was overestimated in the male subgroup (BF%_DXA_< 15%), compared to BF%_DXA_ by 0.45%, respectively. In the other subgroups, the BF%_BIA8_ values were all underestimated. Standing BIA estimating body fat percentage in Chinese participants have a high correlation, but underestimated on normal and high obesity degree in both male and female subjects.

## Background

The prevalence of overweight and obesity has increased tremendously in the global population [1]. Obesity is defined as the over accumulation of body fat and correlates to a risk of high blood pressure, heart problems and diabetes [2]. Thus, periodic assessments of percentage body fat (BF%) may provide valuable information to monitor public health that is quick, low cost, non-invasive and accurate. Many assessment methods can be used to determine BF%, such as the underwater weighing method [3], air-displacement plethysmography and dual energy X-ray absorptiometry (DXA) [4, 5]. However, the application of these methods is limited by their cost and complexity. Therefore, more convenient methods, such as bioelectrical impedance analysis (BIA) and the skinfold method are widely used to assess large populations [6].

In recent years, the measurement protocol of BIA has changed from the traditional supine position with disposable contact electrodes to the standing up position with reusable stainless steel plates as electrodes [7, 8]. Most standing BIA systems operate on a digital scale, and while assessing impedance, the system simultaneously measures the subject's weight through the weight transducer. Measuring body weight during the BIA obtained more accurate estimates than using a self-reported body weight to assess body composition.

Several studies have compared the BF% results as assessed by BIA with other referenced methods [7–11], and the results have been inconclusive. Some concluded that BIA overestimates BF% and some concluded that it underestimates [12, 13]; some concluded that BIA lacks precision while others concluded that it measures with accuracy [10, 14–18]. Questions arise regarding the degree of measurement bias in BF% compared to the reference DXA methods in a healthy population. Furthermore, there is a need for gathering mass quantitative BF% data as limited validation studies exist in the Asian or Chinese population. In the study, we hypothesized that the standing BIA is an accurate method for evaluating body fat percentage in Chinese healthy adult population.

We further compared the differences between the sexes and among different adiposity level subgroups to verify whether the BIA measurement is biased by the levels of adiposity of the Chinese subjects.

## Materials and Methods

### Subjects

Test subjects were selected by a non-random purposive sampling method. The 711 subjects were recruited voluntarily from different locations in Taiwan through advertisements. The subjects were asked to complete health history questionnaires, including personal information, physical characteristics and health conditions. The subjects were further asked to refrain from alcoholic drinks 48 hours prior to the test, from diuretics 7 days prior, and from strenuous physical activities 24 hours prior. Subjects were to void urinary bladder and after a fast > 1.5 h to the experiment. Health questionnaires were distributed to all participants and no test subjects reported any endocrine disorder, nutritional or growth disorders or major chronic conditions, such as diabetes, cancer, kidney dysfunction, asthma and electronic implants, such as an artificial heart or electrodes. Female participants were excluded from measurements if pregnant or during menstruation cycle. The measurements were carried out in the Taichung County Dali Jen Ai Hospital, radiology department. The experimental procedure and research plan were approved by the board of clinical trials of the Jen Ai Hospital (IRB 97–01). All subjects were recruited and signed an informed consent before participating in the study.

### Anthropometry

Each subject was weighed using a Tanita BC-418 (Tanita Co., Tokyo, Japan, BIA_8_ denoted in text) to the nearest 0.1 kg. Subjects’ height was measured without shoes by a Stadiometer to the nearest 0.5 cm and body mass index (BMI) was calculated was calculated as weight divided by height squared (kg/m^2^). The intraexaminer coefficient of variation was 3.6%.

### Measurements of percentage body fat

The subjects wore light cotton robes and removed all metallic objects from their bodies. Body composition parameters, such as total body fat, fat-free soft tissue and bone mineral content, were measured by DXA (Lunar prodigy; GE medical System, Madison, WI). BF% was calculated as fat mass / (fat mass + fat free mass) × 100%. The fat-free mass (FFM) is the sum of the measured total fat-free tissue mass and the bone mineral content. DXA was completed by the Encore 2003 Version 7.0 analytical software. DXA measurements were taken at 2:00 pm each day, and once the DXA measurements were completed, BIA was conducted immediately afterwards. All DXA and BIA_8_ examinations were performed by the same investigator. The intraexamination coefficient of variation for DXA, BIA_8_ was 2% and 2.5%.

We used the BIA_8_ to measure the impedances of the body and of each limb in the standing position. Subjects were asked to hold an electrode shaped like a hand grip in each hand and to stand on base plate electrodes. The electrodes allowed a current to pass through the subject's body and impedance was further measured. The impedances were measured via the pathway from the left foot to the left hand. These impedance measurements were used to estimate the BF%, adjusting for other physical parameters, such as height, weight, age and sex. The BF%, as measured by the DXA and BIA methods, was expressed as BF%_DXA_ and BF%_BIA8_, respectively.

### Statistical analysis

In this study, values are expressed as the mean ± SDs. The paired *t*-test was used to compare the difference in BF%_BIA8_ and BF%_DXA_, Pearson’s correlation, Lin’s concordance correlation coefficient (*ρ*_*c*_) [19] and ordinary least products regression analysis was used to examine the relationship between BF%_BIA8_ and BF%_DXA_ [20]. Statistical significance was set at *P* < 0.05. The Bland-Altman analysis was used to test the agreement between BF%_BIA8_ and BF%_DXA_ [21]. Additionally, one-way ANOVA was used to compare the differences between BF%_BIA8_ and BF%_DXA_ within the different BF% subgroups. We categorised subjects according to the measured adiposity level into: lean, normal and obese categories [22]. Scatter plots for the total, male, and female participants were produced using the BF%_DXA_ as the x-axis and FFM_BIA8_ –FFM_DXA_ as the y-axis, and regression analysis was conducted. All statistical analyses were conducted using SPSS for Windows (Version 17.0; SPSS Inc, Chicago) and Medcalc (Version 11.5; Medcalc Software, Mariakerke, Belgium).

## Results

### Physical characteristics of the subjects

The physical characteristics of the subjects are listed in Table 1. A total of 711 subjects were tested, 412 males and 299 females. The subjects' ages ranged from 18 to 82 years old, with matched age distribution in both sexes. The average male weight exceeded that of females by 14.6 kg, and the height of males exceeded that of females by 13.1 cm. The BMI reported from the subjects ranged from 15.8 to 42.7 kg/m^2^.

**Table 1:** Physical characteristics of the subjects^1^.

	All subjects (*n* = 711)	Male (*n* = 412)	Female (*n* = 299)
Age (y)	34.99 ± 16.64 (18, 82)	33.18 ± 16.89 (18, 82)	37.49 ± 15.98 (18, 78)^2^
Weight (kg)	68.53 ± 14.60 (38, 133)	74.65 ± 13.07 (42, 133)	60.09 ± 12.22 (38, 108)^3^
Height (cm)	167.27 ± 9.73 (145, 200)	172.76 ± 7.61 (152,200)	159.70 ± 6.83 (143, 181)^3^
BMI (kg/m^2^)	24.38 ± 4.12 (15.8, 42.7)	24.96 ± 3.71 (16.8,41.8)	23.57 ± 4.51 (15.8, 42.7)^2^

^1^ All values are mean ± SDs; minimum and maximum in parentheses.

^2,3^ Significantly different from male (one-factor ANOVA); ^2^*P* < 0.05, ^3^*P* < 0.001.

### Comparison of BF% measured by BIA and DXA

**Fig 1:**
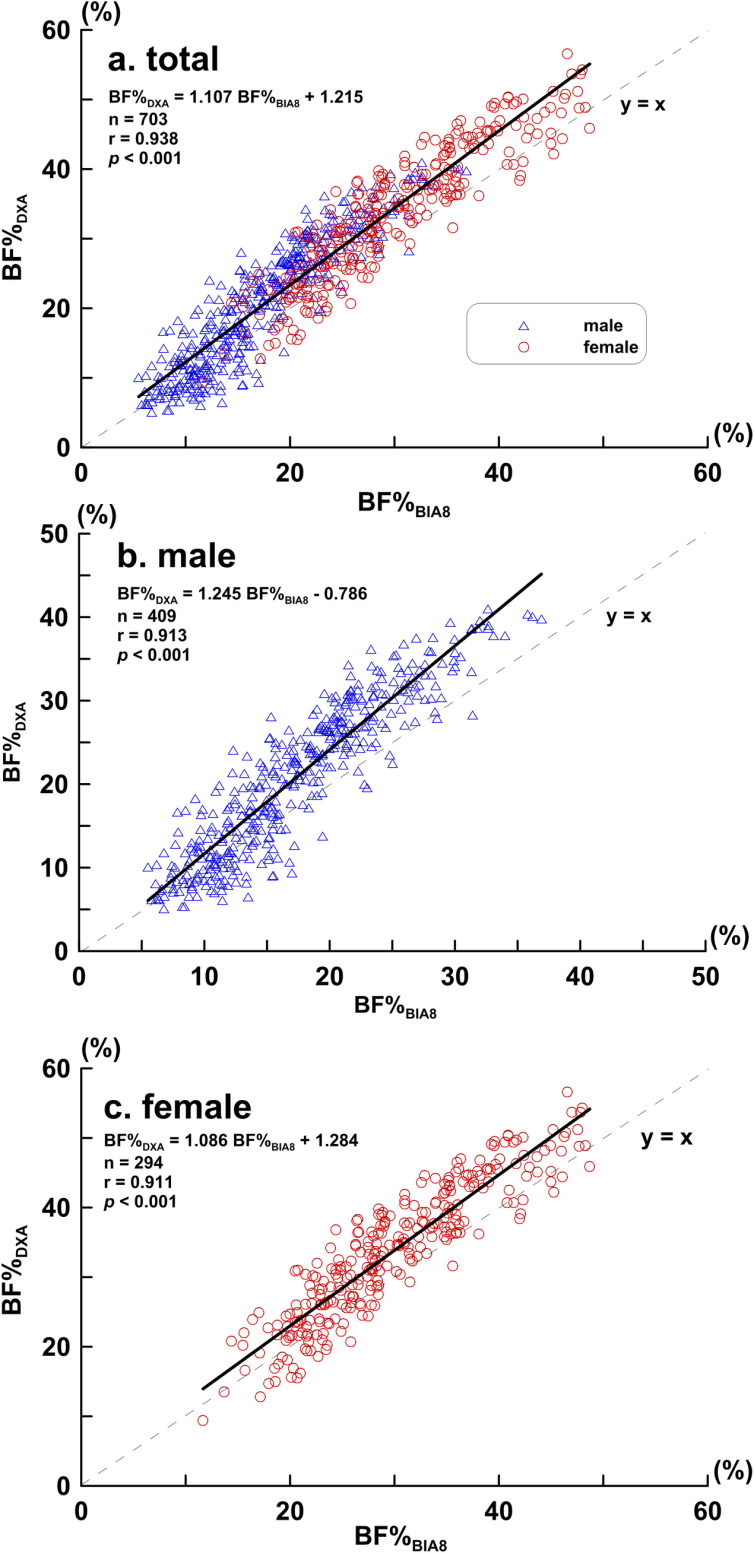
**BF%**_**BIA8**_ **and BF%**_**DXA**_ **scatter plot and regression line (a) all participants (b) male subjects (C) female subjects; Bold line represents regression line, dotted line represents identical line**.

The subjects' BF% measured by BIA_8_ and DXA are listed in Table 2. The average BF%_BIA8_ and BF%_DXA_ were 22.54 ± 9.48% and 26.26 ± 11.18% respectively. The measured BF%_BIA8_ was significantly lower than BF%_DXA_ in both male and female subjects. The correlation coefficient between BF%_BIA_ and BF%_DXA_ for all subjects was calculated as 0.93, while for the male and female subgroups, they were 0.90 and 0.92, respectively. (Fig 1)

**Table 2:** Percentage body fat measured by standing-posture bioelectrical impedance analysis (BIA) and by dual-energy X-ray absorptiometry (DXA)^1^.

Method	All subjects (*n* = 711)	Male (*n* = 412)	Female (*n* = 299)
BF%_BIA8_	22.54 ± 9.48 (5.5, 48.7)^2^	17.24 ± 6.53(5.5, 36.9)^2^	29.85 ± 7.93(11.7, 48.7)^2^
BF%_DXA_	26.26 ± 11.18 (5.1, 56.6)	20.89 ± 9.05 (5.1,41.0)	33.66 ± 9.49(10.6, 56.6)

^1^ All values are mean ± SDs; minimum and maximum in parentheses.

^2^ Significantly different from DXA, *P* < 0.001 (paired *t*-test).

### BF%_BIA8_ bias on the basis of BF%_DXA_

**Fig 2:**
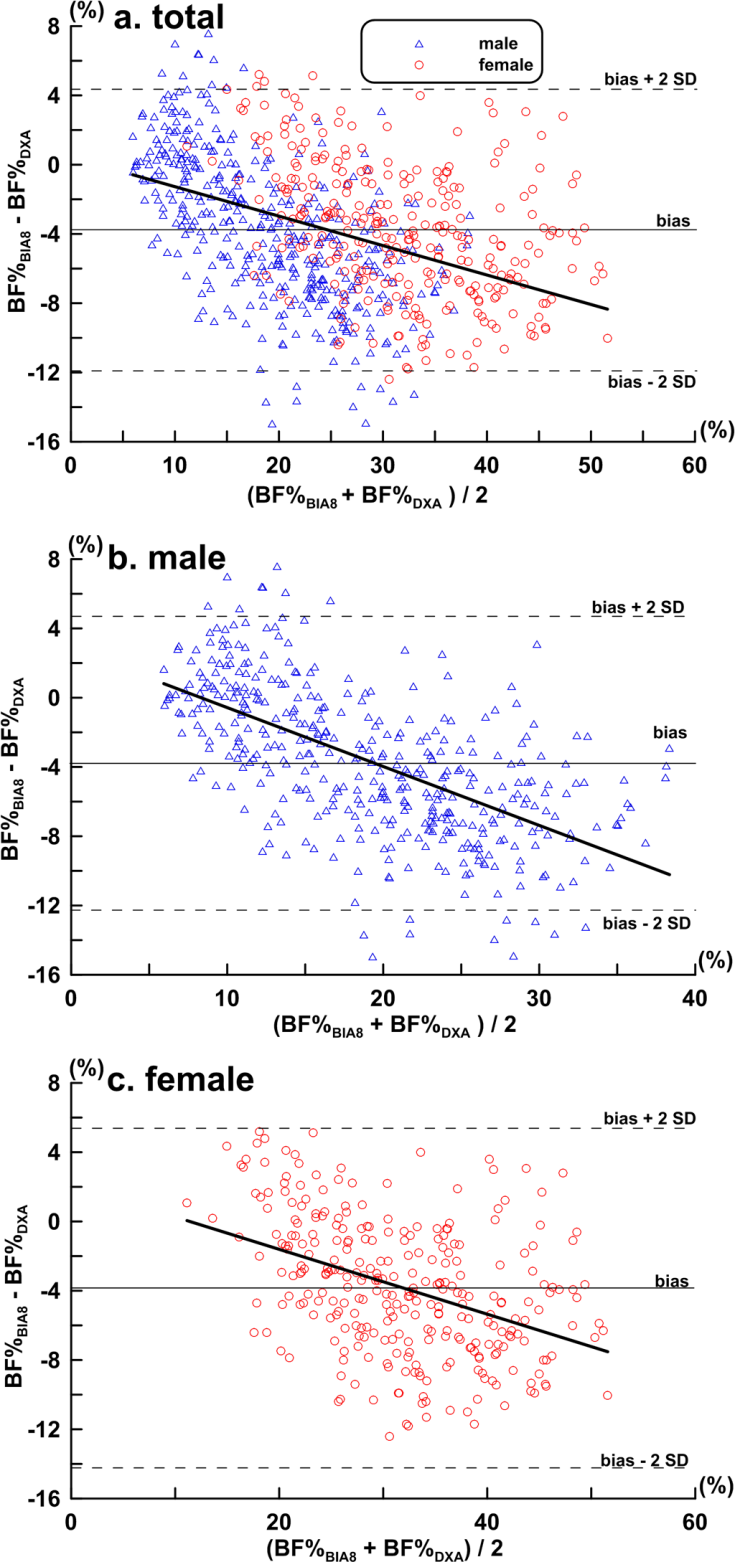
Bland-Altman plot of the difference between BF%_BIA8_ and BF%_DXA_ in mean difference expressed as bias, 95% confidence interval expressed as bias ± 2 SD. (a) Total subjects (*n* = 711); bias ± SD: -3.72 ± 4.09%, bias– 2SD: -11.90%, bias + 2 SD: 4.46%, regression equation y = - 0.170 x + 0.430 (*r* = 0.42, *P* < 0.01); (b) Male (*n* = 412); bias ± SD: -3.66 ± 4.24%, bias– 2SD: -12.14%, bias + 2 SD: 4.83%, regression equation y = - 0.340 x + 2.830(*r* = 0.61, *P* < 0.01); (c) Female (*n* = 299); bias ± SD: -3.81 ± 3.87%, bias– 2SD: -11.56%, bias + 2 SD: 3.94%, regression equation y = - 0.187 x +2.134 (*r* = 0.41, *P* < 0.01).

**Fig 3:**
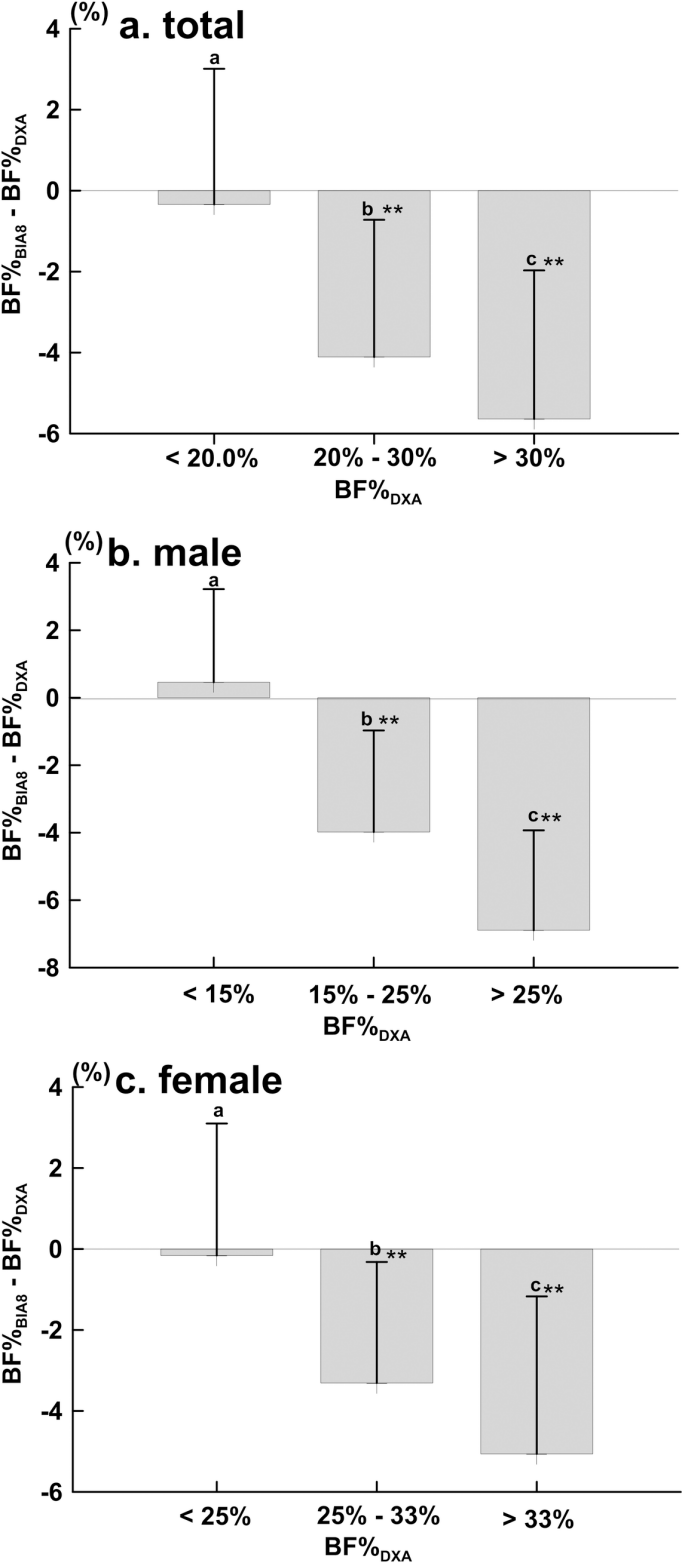
**BF% dependent bias of BIA**_**8**_ **compared with DXA in (a) total (*n* = 711), (b) male (*n* = 412), and (c) female (*n* = 299).** Data are presented as the mean difference ± SD. Means with symbol are significantly different, *P*< 0.001 (**).

The Bland-Altman analysis was used to test the agreement between BF%_DXA_ and BF%_BIA8_ by dividing all male and female subjects in two different ways: (i) uncategorised, including all subjects, (ii) categorised into lean, normal and obese subgroups. The results are shown in Fig 2(A), 2(B) and 2(C), Fig 3(A), 3(B) and 3(C) respectively.

Fig 2(A) presents the results of the Bland-Altman analysis between the BF%_DXA_ and BF%_BIA8_ across all subjects, and the bias ± SD between the two results was -3.72 ± 4.09%. Fig 3(A) presents the results when subjects were categorised into lean—BF%_DXA_: <20% (*n* = 212), normal—BF%_DXA_: 20%-30% (*n* = 229) and obese—BF%_DXA_: >30% (*n* = 270) subgroups. BF%_BIA8_ underestimated BF % compared to BF%_DXA_ in the lean, normal and obese subgroups by -0.40± 3.31%, -4.13 ± 3.42% and -5.98 ± 3.42%, respectively.

Fig 2(B) presents the results of the Bland-Altman analysis between BF%_DXA_ and BF%_BIA8_ across all male (*n* = 412) subjects, and the bias ± SD between the two results was -3.66 ± 4.24%. Fig 3(B) presents the results when the male subjects were divided into lean- BF%_DXA_:< 15% (*n* = 135), normal- BF%_DXA_: 15%-25% (*n* = 129) and obese- BF%_DXA_:>25% (*n* = 148) subgroups. BF%_BIA8_ overestimated BF% compared to BF% _DXA_ in the lean subgroups by 0.45± 2.76%, but it underestimated BF% in the normal and obese subgroup by -4.01 ± 2.77% and -7.01 ± 3.11%, respectively.

Fig 2(C) presents the results of the Bland-Altman analysis between BF%_DXA_ and BF%_BIA8_ across all female subjects (*n* = 299), and the bias ± SD between the two results was -3.81 ± 3.87%. Fig 3(C) presents the results when all female subjects were categorised into lean -BF%_DXA_: < 25% (*n* = 64), normal—BF%_DXA_: 25%–33% (*n* = 78) and obese -BF%_DXA_ > 33% (*n* = 157) subgroups. BF%_BIA8_ underestimated BF% compared to BF%_DXA_ in lean, normal and obese subgroups by -0.15± 3.23%, -3.35 ± 2.91% and -5.53 ± 3.42%, respectively.

When focus on the different levels of adiposity subgroups in male and female subjects and applying linear regression analysis to the data, the result showed no fixed and proportional bias from the slope and intercept 95%CI between BF%_BIA8_ and BF%_DXA_ in male and female subject. Other subgroup all showed proportional and/or fixed bias (Table 3). Lin’s concordance correlation coefficients (*ρ*_*c*_) are shown in Table 3. McBride [23] suggests the following descriptive scale for values of the *ρ*_*c*_: Value of *ρ*_*c*_ < 0.90 is poor and 0.90 to 0.95 is moderate. In Table 3, the concordance between the these two methods was poor for all of the *ρ*_*c*_ value were less than 0.09.

**Table 3:** Body fat percentage outcome of analyses by ordinary least products regression.

Proportional^1^	*r*	*a*	95%CI	*b*	95%CI	Proportional bias	Fixed bias	*ρ*_*c*_
(a) total	0.933	1.609	0.829, 2.389	1.083	1.051, 1.115	Yes	Yes	0.874
(b) male	0.902	-0.710	-1.802, 0.382	1.243	1.183, 1.303	Yes	No	0.782
(c) female	0.910	1.916	0.144, 3.688	1.053	0.996, 1.110	No	Yes	0.837
(c) total_lean_	0.654	4.482	3.072, 5.892	0.674	0.568, 0.780	Yes	Yes	0.850
(d) total_normal_	0.479	17.778	15.935, 19.620	0.346	0.259, 0.433	Yes	Yes	0.676
(e) total_obese_	0.857	14.263	12.426, 16.111	0.732	0.676, 0.788	Yes	Yes	0.687
(f) male_lean_	0.488	5.636	4.092, 7.178	0.442	0.305, 0.579	Yes	Yes	0.480
(g) male_normal_	0.581	12.343	10.265, 14.421	0.492	0.369, 0.615	Yes	Yes	0.327
(h) male_obese_	0.777	14.183	11.794, 16.571	0.695	0.597, 0.793	Yes	Yes	0.344
(i) female_lean_	0.491	9.580	4.527, 14.632	0.539	0.295, 0.784	Yes	Yes	0.489
(j) female_normal_	0.408	21.277	16.995, 25.559	0.306	0.141, 0.470	Yes	Yes	0.223
(k) female_obese_	0.791	16.117	12.835, 19.399	0.694	0.604, 0.783	Yes	Yes	0.572

^1^ Relationship between different subgroups in BF%_DXA_ and BF%_BIA8_, lean, normal, obese subscripts represents DXA measured results; *r*, product-moment correlation coefficient; a, b, coefficients in ordinary least products regression model E(A) = *a* + *b*(B); *a*, A (y axis) intercept; *b*, slope; proportional bias, if 95% confidence interval (CI) for *b* does not include 1; fixed bias, if 95% CI for a does not include; Lin’s concordance correlation coefficient (*ρ*_*c*_).

**Fig 4:**
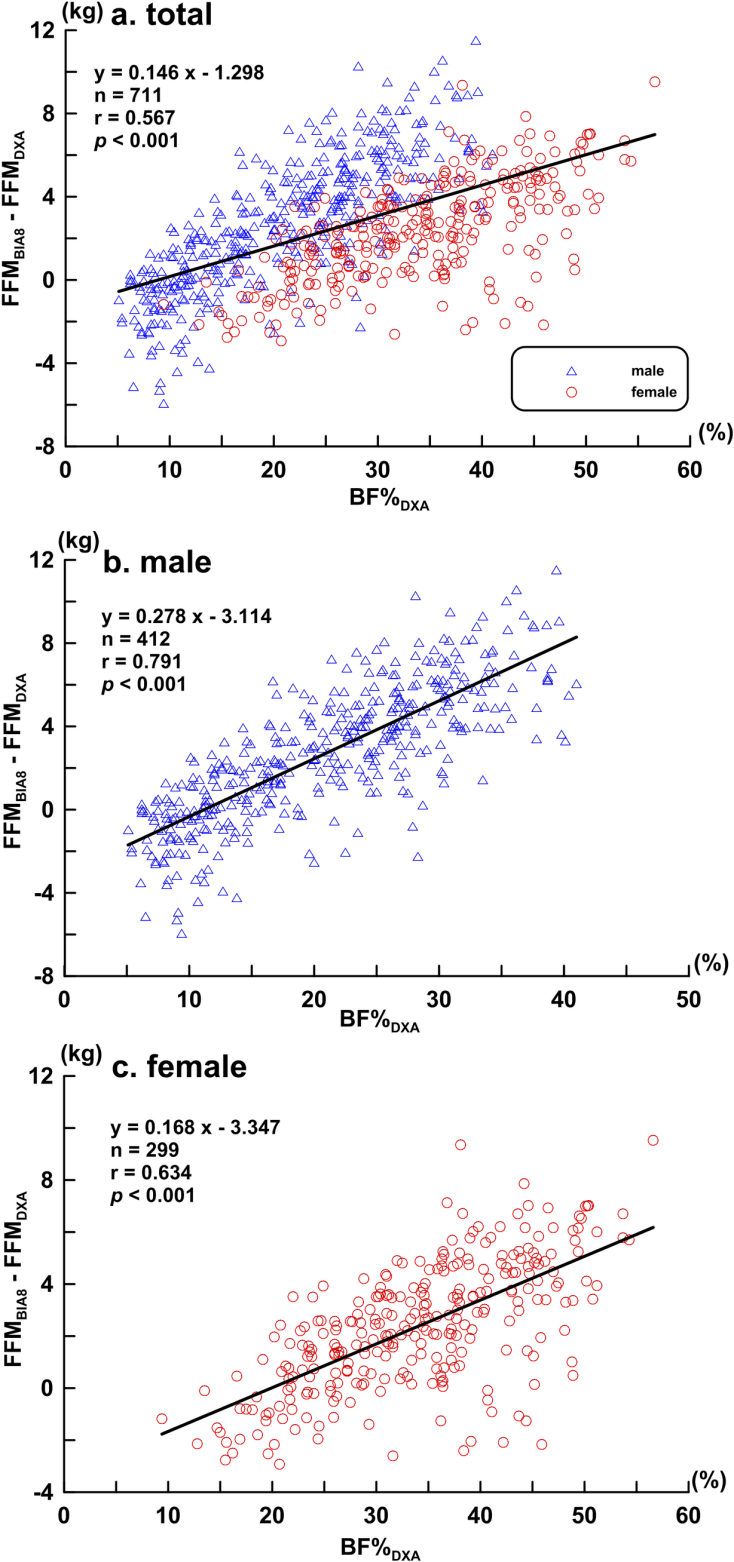
Scatter and regression plots of BF%_DXA_ according to FFM_BIA8_ and FFM_DXA_ differences. **(a) total subjects (*n* = 711), (b) male subjects (*n* = 412), and (c) female subjects (*n* = 299).** The bold line represents the regression line.

Fig 4 shows the scatter plot and regression analyses of the BF%_DXA_ according to FFM_BIA8_ and FFM_DXA_ differences.

## Discussion

The use of Tanita BC-418 for estimating and validating body composition has been reported in numerous studies [13, 16, 18, 24–26], but studies have only utilized a relatively smaller sample size to validate the accuracy of BIA. Hence, the present study compared the measured BF% by both the BIA_8_ and DXA methods in a large sample of a healthy Chinese population in Taiwan. The results of the study showed that using BIA_8_ to estimate BF% yields results that are similar to those of the DXA method in both male and female subjects. Sun *et al*. compared the BF%_DXA_ with an estimated BF% in a supine posture BIA with multiple frequencies in a large healthy Canadian population and attained similar results. However, according to the results of our study, BF%_BIA8_ had a higher correlation with BF%_DXA_ than BF% did when estimated by a supine posture BIA [9].

Body composition information can be applied in clinical trials and other medicine-related fields [27, 28]. Some assessment methods, such as DXA, air displacement plethysmography and underwater weighing, can provide accurate estimates, but these methods are often expensive and cannot be widely applied to the general public. BIA is a feasible alternative for practical use in assessing a large-scale sample. Existing BIA studies have shown conflicted and inconsistent results regarding the accuracy of BF% estimation by BIA. To clarify these results, our study used four different approaches: 1. the sample size was 711, larger than that of previous BIA-related research; 2. the sample had a wider age-range with even age distribution in male and female subjects; 3. we grouped subjects into different BF% categories, compared the results of the measured BF% by BIA_8_ against that by DXA and examined the trend line between the two measurements; and 4. Use different genders, obesity level, analysing the DXA proportional bias and fixed bias results.

The manufacturers of the currently available BIA measuring equipment have not released their built-in prediction equations or identified a suitable testing population, which limits the reference and application value of BIA [14]. This work used the raw data obtained from the Tanita BC-418 model instead of using the built-in prediction equations for estimating BF%.

This research shows that a high degree of correlation exists between BF%_BIA8_ and BF%_DXA_, which is consistent with other research [13, 17]. A high correlation is shown in both the male and the female subgroups; however, testing of the correlation alone may not guarantee the equivalence of the two measuring methods [20, 21]. After comparison of the differences between BF%_BIA8_ and BF%_DXA_ by the Bland-Altman analysis, the results show that for mixed, male and female sample sets, BF%_BIA8_ underestimates BF% compared to BF%_DXA_, but the differences do not deviate with statistical significance (male: -3.66 ± 4.24%, female: -3.81 ± 3.87%).

The Bland-Altman analysis is usually used to explore variables without categorising or splitting the sample so that the agreement between two measurement methods may be analysed. However, this research examined the distributions and trends of the measured differences not only by splitting the sample into male and female subgroups but also by categorising them according to their measured BF%_DXA_. Sun *et al*. used the Bland-Altman analysis to investigate the differences between supine posture BIA and DXA measurements of BF% according to sex and adiposity, and they also categorised subjects into total, male and female, as well as lean, normal and obese [9]. Although research has tested the differences between BIA and DXA by lean, normal, obese criteria, further comparison of the two methods by other criteria may be necessary to fully understand the differences in the measurements. As for the lean, normal, obese subgroups in the male, female and combined subgroups, BF% was still underestimated by BIA_8_ compared to DXA, and the degree of the bias also increased as the BF%_DXA_ increased. These results are consistent with the BF% of obese women measured by the Tanita BC-418, as reported by Neovius *et al*. [16] Studied male and female participants’ obesity level using Tanita BC-418 and comparing with DXA measured results and showed that BF% has been underestimated. Further, the present study also showed consistent result as reported by Hemmingsson, Mally, Lee *et al*. used different race, age and obesity level to estimate BF% between Tanita BC-418 and DXA measurements. At the end, the authors reported that BC-418 have underestimated BF% than of DXA measured results [24–26].

Oshima *et al*. stated that a standing hand-to-foot BIA is a stable and suitable method for assessing whole body BF%, and the variation of within-day impedance measurements determined by standing hand-to-foot BIA was smaller than the variation of those determined by standing hand-to-hand or foot-to-foot BIAs [29]. Additionally, BIA methods generated more accurate results, compared to foot-to-foot BIA [30].

A distinct measuring difference was found when it comes to the DXA apparatus designed by different manufactories. The evaluated BF% data measured from the Hologic QDR series were always higher than that of Lunar DPX series [31]. In lower adiposity level subjects, the evaluated BF% measured from Lunar Expert was lower than Hologic QDR4500, but in high adiposity level subjects, Lunar Expert had a higher BF% than Hologic QDR4500 [32]. Furthermore, based on the DXA BF% measuring results, we conclude that with different DXA apparatus and adiposity levels will affect the measuring results of the study.

This study used parallel measurements of BF% by hand-to-foot BIA_8_ and DXA to examine the measurement differences with in various BF% subgroups. In both male and female subjects except for subjects with adiposity lower than normal levels, BIA_8_ has the tendency to underestimate BF%. These differences increase with the level of adiposity; BIA_8_ overestimated BF% in the low BF% group and underestimated BF% in the high BF% group. These findings are similar to the trend reported by previous supine posture BIA study [9]. In the present study, a high correlation was found for evaluating BF% between standing BIA and DXA. The standing BIA estimated results was significantly underestimated, therefore we reject the hypothesis for the present study.

## Conclusions

In summary, BIA_8_ may generate inaccurate measurements when subjects with high adiposity and when applying measurements to the Chinese population, as participants’ adiposity levels increases, BF% will increase its underestimation correspondingly.

## Abbreviations

BF%: Body fat percentage
BIA: Bioelectrical impedance analysis
BMI: Body mass index
CI: Confidence interval
DXA: dual-energy X-ray absorptiometry
FFM: Fat free mass
SD: standard deviations
